# Endogenous hydrogen sulfide is associated with angiotensin II type 1 receptor in a rat model of carbon tetrachloride-induced hepatic fibrosis

**DOI:** 10.3892/mmr.2015.3873

**Published:** 2015-06-02

**Authors:** HUI-NING FAN, NI-WEI CHEN, WEI-LIN SHEN, XIANG-YUN ZHAO, JING ZHANG

**Affiliations:** Department of Gastroenterology, Shanghai Jiaotong University Affiliated Sixth People's Hospital, Shanghai 200233, P.R. China

**Keywords:** DL-propargylglycine, sodium hydrosulfide, carbon tetrachloride, hepatic fibrosis, angiotensin II type 1, hydrogen sulfide, cystathionine-β-synthase

## Abstract

The present study aimed to investigate the effects of endogenous hydrogen sulfide (H_2_S) on the expression levels of angiotensin II type 1 receptor (AGTR1) in a rat model of carbon tetrachloride (CCl_4_)-induced hepatic fibrosis. A total of 56 Wistar rats were randomly divided into four groups: Normal control group, model group, sodium hydrosulfide (NaHS) group, and DL-propargylglycine (PAG) group. Hepatic fibrosis was induced by CCl_4_. The rats in the PAG group were intraperitoneally injected with PAG, an inhibitor of cystathionine-γ-lyase (CSE). The rats in the NaHS group were intraperitoneally injected with NaHS. An equal volume of saline solution was intraperitoneally injected into both the control and model groups. All rats were sacrificed at week three or four following treatment. The serum levels of hyaluronidase (HA), laminin protein (LN), procollagen III (PcIII), and collagen IV (cIV) were detected using ELISA. The serum levels of alanine transaminase (ALT), aspartate transaminase (AST), and albumin (ALB) were detected using an automatic biochemical analyzer. The liver mRNA expression levels of CSE were detected by reverse transcription-quantitative polymerase chain reaction. The liver expression levels of AGTR1 and the plasma expression levels of H_2_S were detected using western blot analyses. The results indicated that the severity of hepatic fibrosis, the serum expression levels of HA, LN, PcIII, cIV, ALT, and AST, the liver expression levels of CSE and AGTR1, and the plasma expression levels of H_2_S were significantly higher in the PAG group, as compared with the model group (P<0.05). Conversely, the expression levels of ALB were significantly lower in the PAG group, as compared with the model group. In addition, the severity of hepatic fibrosis, the serum expression levels of HA, LN, PcIII, cIV, ALT, and AST, the liver expression levels of CSE and AGTR1, and the plasma expression levels of H_2_S were significantly lower in the NaHS group, as compared with the model group (P<0.05). These results suggest that endogenous H_2_S is associated with CCl_4_-induced hepatic fibrosis in rats, and may exhibit anti-fibrotic effects. Furthermore, H_2_S reduced the liver expression levels of AGTR1, which may be associated with the delayed progression of hepatic fibrosis.

## Introduction

Hydrogen sulfide (H_2_S) has previously been identified as a signaling molecule that exhibits numerous physiological and pathological activities (1). Exogenous sodium hydrosulfide (NaHS) releases H_2_S, in order to induce physiological responses. Previous studies have demonstrated that H_2_S may relax vascular and ileal smooth muscle in the cardiovascular system (2), increase colonic secretion and reduce gastric injury in the gastrointestinal system (3), attenuate neuronal injury (4), and prevent the development of hypertension (5). The roles of H_2_S also include the inhibition of oxidative stress (6), the production of lipid peroxidation and inflammatory factors (7), and the activation of ATP-sensitive potassium channels (K_ATP_) (8).

H_2_S regulates hepatic-related injury, and is produced in the liver by both cystathionine-γ-lyase (CSE) and cystathionine-β-synthase (CBS), which use L-cysteine as a substrate to produce H_2_S. H_2_S exhibits anti-inflammatory and cytoprotective activities against hepatic ischemia reperfusion (I/R) injury (9). Carbon tetrachloride (CCl_4_)-induced downregulation of H_2_S production and CSE expression is associated with the development of increased intrahepatic resistance, portal hypertension, and hepatic fibrosis in a rat model of liver cirrhosis (10). A previous study suggested that H_2_S and CSE exhibit anti-fibrotic effects in pulmonary fibrosis (11). However, to the best of our knowledge, there have been few attempts to investigate the role of H_2_S in hepatic fibrosis.

Hepatic fibrosis is a common response to chronic liver injury caused by various diseases. The mechanisms underlying the development of hepatic fibrosis consist predominantly of the activation of hepatic stellate cell (HSCs), and the accumulation of extracellular matrix components within the liver (12). The renin angiotensin system (RAS) is involved in the pathogenesis of fibrosis, both in the heart and various other organs (13,14). Notably, activated human HSCs express RAS components and synthesize angiotensin II. Furthermore, angiotensin II type 1 receptors (AGTR1) are located in HSCs (15). Previous studies have demonstrated that the control of RAS activation by AGTR1 antagonists may have therapeutic potential in the treatment of hepatic fibrosis. H_2_S and NaHS decrease AGTR1 binding, as well as AGTR1 binding affinity in spontaneously hypertensive rats (16). The present study aimed to investigate whether H_2_S affects hepatic fibrosis by regulating the expression of AGTR1.

## Materials and methods

### Materials and reagents

Pathogen-free male Wistar rats (weighing 200–300 g) were purchased from XiPuer-Rubicam Experimental Animals, Ltd. (Shanghai, China). Analytical grade CCl_4_ and DL-propargylglycine (PAG) were purchased from Beijing Dingguo Changsheng Biotechnology Co., Ltd. (Beijing, China). Analytical grade zinc cetate, N,N-dimethy-p-phenylenediamine, HCl, trichloroacetic acid, and NaHS were purchased from Sigma-Aldrich (St. Louis, MO, USA). The rat ELISA kit was purchased from Sigma-Aldrich, the reverse transcription-quantitative polymerase chain reaction (RT-qPCR) kit was obtained from Takara Bio, Inc. (Tokyo, Japan), and the Western Blotting kit was from Abcam (Cambridge, UK). The current study was approved by the ethics committee of Shanghai Jiaotong University Affiliated Sixth People's Hospital (Shanghai, China).

### Experimental design

The rats were randomly divided into four groups (n=14/group): Normal control group, model hepatic fibrosis group, NaHS group, and PAG group. NaHS is a H_2_S donor, and PAG is a CBS inhibitor. Each group was then randomly divided into two subgroups each containing seven rats; one of the subgroups received treatment for three weeks, whereas the other one received treatment for four weeks. The rats were maintained in a sterile environment with *ad libitum* access to drinking water, and underwent a 12 h light/dark cycle. Hepatic fibrosis was induced using 5 ml/kg 40% CCl_4_ in corn oil tree time weekly for three or four weeks in all groups, except for the normal control group. The rats in the PAG group were intraperitoneally injected with 45 *µ*mol/kg/day PAG, a CBS inhibitor. The rats in the NaHS group were intraperitone-ally injected with 56 *µ*mol/kg/day NaHS, H_2_S donor. An equal volume of saline was intraperitoneally injected into the control and model group rats. The rats were then sacrificed with 2% pentobarbital (H. Lundbeck Co., Copenhagen, Denmark) at week three or four following modeling, depending on their subgroup.

### Serum biochemical measurements

The serum expression levels of alanine aminotransferase (ALT), aspartate aminotransferase (AST), and albumin (ALB) were determined using commercially available kits (Diatech Diagnostics, Hungary) using the Autobiochemical Analyzer (Toshiba, Tokyo, Japan) following centrifugation at 3,000 × g at 4°C of the blood collected at 4,000 rpm for 15 min. The serum expression levels of hyaluronidase (HA), laminin protein (LN), procollagen III (PcIII), and collagen IV (cIV) were measured using an ELISA kit.

### Histopathological examination

The livers were removed and washed with saline to remove excess blood. Liver tissue sections (5 *µ*m) were fixed in formalin and embedded in paraffin prior to examination. The liver sections were then stained using hematoxylin and eosin (HE) and Masson's trichrome (Wuhan Biotechnology Ltd., Co., Wuhan, China), in order to determine the stage of hepatic fibrosis. The fibrotic stages were scored according to Brunt (17): S0, no fibrosis; S1, portal fibrous expansion; S2, thin fibrous septa emanating from portal triads; S3, fibrous septa bridging portal triads and central veins; S4, cirrhosis.

### RT-qPCR

RT-qPCR was performed in order to analyze the mRNA expression levels of CSE in hepatic tissue, as previously described (18). Total RNA (2 *µ*g) was extracted from the hepatic samples and reverse transcribed using the RevertAid First-Strand cDNA Synthesis kit (Thermo Fisher Scientific, Inc., Waltham, MA, USA), according to the manufacturer's instructions. The PCR primer sequences from Takara Bio, Inc. (Tokyo, Japan) were as follows: CSE, forward 5′-CCACCACAACGATTACCCA-3′, reverse 5′-TCAGCACCCAGAGCCAAAG-3′; and β-actin, forward 5′-TCCTGACCCTGAAGTACCCCATTG-3′, and reverse 5′-GGAACCGCTCATTGCCGATAGT-3′. RT-qPCR was performed using the SYBR Green qPCR Super Mixture (Takara Bio, Inc.) and an ABI Prism 7500 Sequence Detection System (Applied Biosystems Life Technologies, Foster City, CA, USA). Amplification was performed with the following cycles: 95°C for 2 min, followed by 40 cycles of denaturing at 95°C for 15 sec, and annealing at 60°C for 32 sec. All reactions were performed in triplicate. Data analysis was performed using the 2^−ΔΔCT^ method, as described by Livak and Schmittgen (19), with β-actin acting as a reference gene.

### Western blot analysis

The protein expression levels of AGTR1 were detected by western blotting. The membrane protein fractions were prepared as previously described (20). Briefly, 100 mg liver tissue samples were first homogenized ultrasonically for 4 min using a Tissue Pulverizer (BioSpec Products, Bartlesville, OK, USA). The samples were then lysed in radioimmunoprecipitation buffer, separated by 10% SDS-PAGE and electro-transferred to nitrocellulose membranes (EMD Millipore, Billerica, MA, USA). The membranes were then blocked with 5% non-fat dry milk in tris-buffered saline containing Tween^®^ 20 [TBST; 10 mmol/l Tris-hydrochloric acid (HCl) pH 8.0, 150 mmol/l NaCl, and 1% Tween^®^ 20]. The membranes were probed with a rabbit polyclonal AGTR1 antibody (cat. no. sc-1173; Santa Cruz Biotechnology, Inc., Dallas, TX, USA) at a dilution of 1:1,000 in blocking solution (10 mM Tris-HCl, 5% powdered milk, 2% bovine serum albumin, 0.1% Tween^®^ 20, pH 7.6; Sigma-Aldrich). Following extensive washing with TBST, the membranes were incubated for 1 h with a secondary horseradish peroxidase (HRP)-conjugated goat anti-rabbit antibody (cat. no. A6154; Sigma-Aldrich) at a dilution of 1:2,000 in phosphate-buffered saline (PBS). After a final washing step with TBST, the blots were visualized using an enhanced chemiluminescence kit (GE Healthcare Life Sciences, Chalfont, UK). In order to detect β-actin, the membranes were stripped with 0.2% SDS, 0.1% mercaptoethanol, and 1 M Tris pH 6.8 for 30 min at 70°C, prior to being incubated with a mouse monoclonal β-actin antibody (cat. no. A5441, Sigma-Aldrich) at a dilution of 1:4,000 in blocking solution for 1 h. The membranes were then incubated with the HRP-conjugated goat anti-rabbit antibody (cat. no. A6154, Sigma-Aldrich) at a dilution of 1:2,000 in PBS for 1 h. The results of the enhanced chemiluminescence analysis were digitized by conventional scanning, and quantified using computerized image analysis software Alpha Imager 2000, according to the manufacturer's instructions. The densitometric results were expressed in relation to the ratio of AGTR1 and β-actin.

### Measurement of H_2_S serum levels

The serum levels of H_2_S were measured as described previously (13). Briefly, 75 *µ*l aliquots of sera were mixed with 100 *µ*l distilled water, and 300 *µ*l 10% trichloroacetic acid. The reaction was terminated by the addition of 150 *µ*l 1% zinc acetate. A total of 20 *µ*M N,N-dimethyl-p-phenylenediamine sulfate in 7.2 M HCl, and FeCl_3_ (30 *µ*M; 133 *µ*l) in 1.2 M HCl was then added to the solution. Following a 15 min incubation at room temperature, the absorbance of the resulting solution was measured using a UV-2550 spectrophotometer at 670 nm (Shimadzu Corporation, Tokyo, Japan). All samples were assayed in triplicate, and the levels of H_2_S were calculated against the NaHS calibration curve (0.122–250 *µ*M).

### Statistical analysis

The results are expressed as the mean ± standard deviation. The data were analyzed by a one-way analysis of variance followed by Newman-Keuls comparisons using SPSS version 17.0 (SPSS, Inc., Chicago, IL, USA). P<0.05 was considered to indicate a statistically significant difference.

## Results

### Effects of H_2_S on CCl_4_-induced hepatic fibrosis

**Figure 1:**
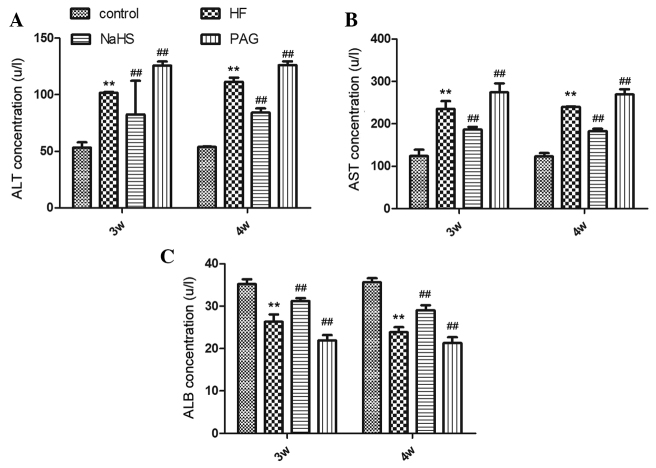
Serum expression levels of (A) alanine transaminase, (B) aspartate transaminase, and (C) albumin for each group. The error bars indicated the mean ± standard deviation (^**^P<0.01 vs. normal control group, ^##^P<0.01 vs. the model group). NaHS, sodium hydrosulfide; PAG, DL-propargylglycine; HF, hepatic fibrosis.

Treatment with NaHS significantly attenuated the CCl_4_-induced serum expression levels of ALT and AST, whereas PAG increased the serum expression levels of AST and ALT. Conversely, the serum expression levels of ALB were decreased in the PAG group, and increased in the NaHS group (Fig. 1). The observed CCl_4_-induced impaired liver function and the protective effects of H_2_S were supported by histological alterations. The liver sections from the normal control group rats exhibited normal histology (Table I), whereas the liver sections from the CCl_4_-induced model group rats exhibited focal necrosis and degeneration. NaHS delayed both necrosis and vacuolization, whereas PAG aggravated hepatic injury and caused increased inflammatory cell infiltration.

### Effects of NaHS and PAG on CCl_4_-induced hepatic fibrosis

**Figure 2:**
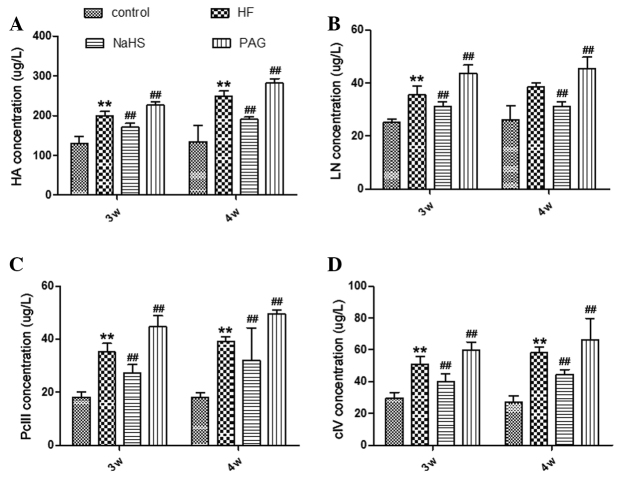
Serum expression levels of (A) hyaluronidase, (B) laminin protein, (C) procollagen III, and (D) collagen IV for each group. The error bars indicated the mean ± standard deviation (^**^P<0.01 vs. normal control group, ^##^P<0.01 vs. the model group). NaHS, sodium hydrosulfide; PAG, DL-propargylglycine; HF, hepatic fibrosis.

**Figure 3:**
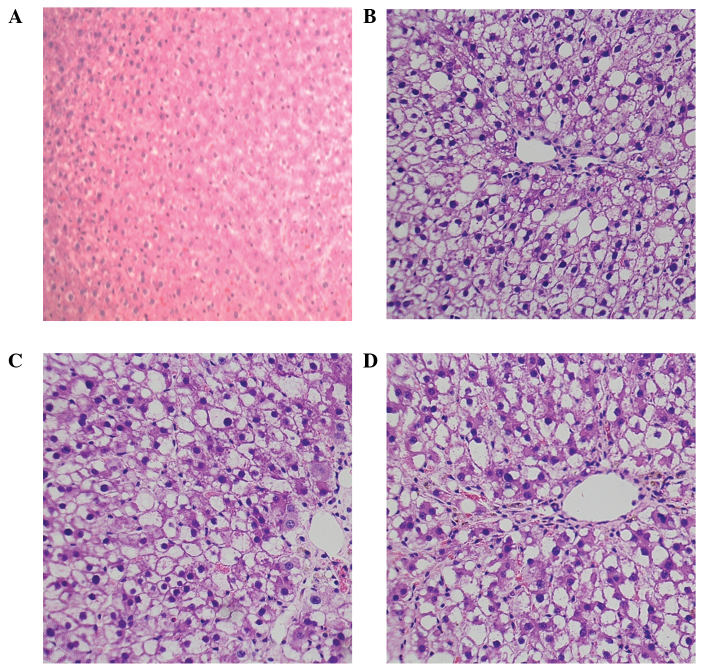
Pathology of the hepatic tissues for each group at week three. The tissues were stained with hematoxylin and eosin (magnification ×200). (A) Control group, (B) model group, (C) sodium hydrosulfide group, (D) DL-propargylglycine.

**Figure 4:**
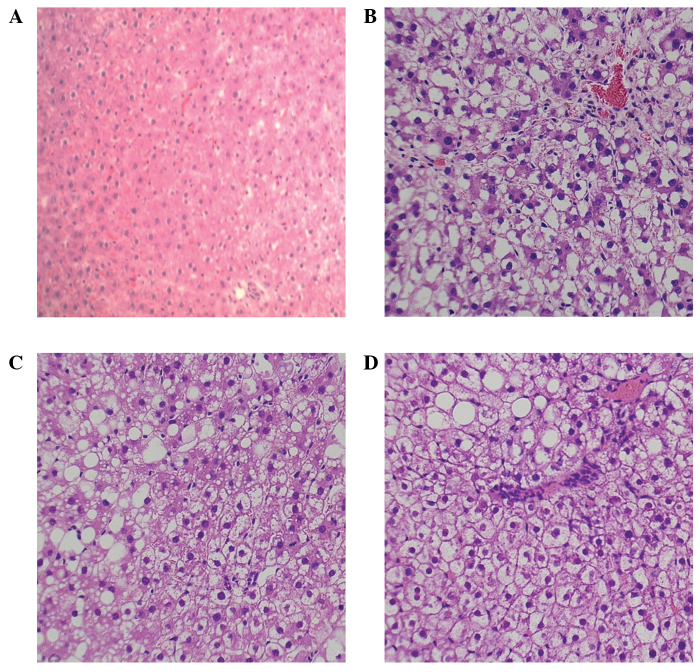
Pathology of the hepatic tissues for each group at week four. The tissues were stained with hematoxylin and eosin (magnification ×200). (A) Control group, (B) model group, (C) sodium hydrosulfide group, (D) DL-propargylglycine.

**Figure 5:**
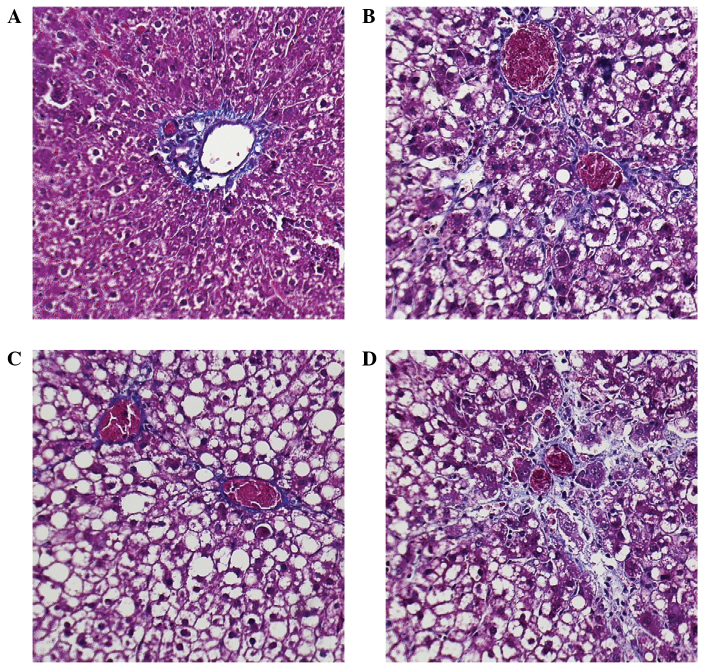
Pathology of the hepatic tissues for each group at week three. The tissues were stained with Masson's trichrome (magnification ×200). (A) Control group, (B) model group, (C) sodium hydrosulfide group, (D) DL-propargylglycine.

**Figure 6:**
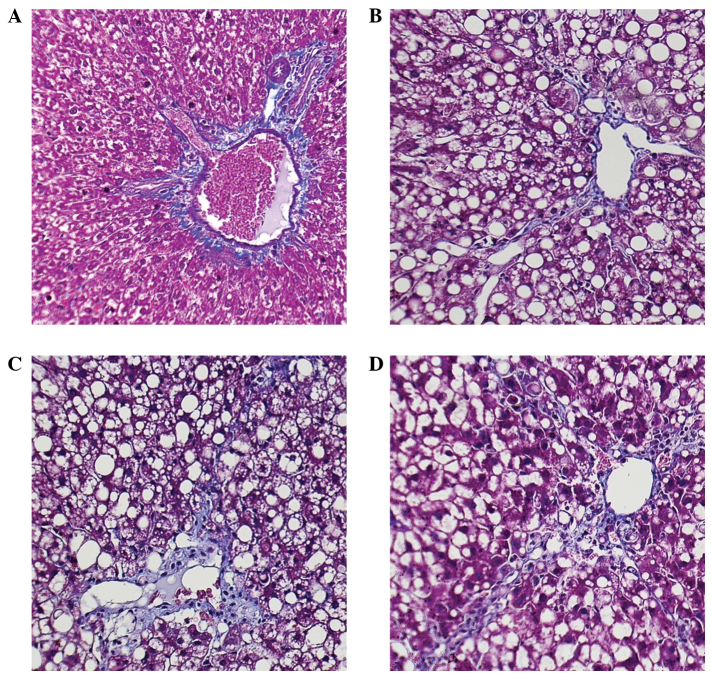
Pathology of the hepatic tissues for each group at week three. The tissues were stained with Masson's trichrome (magnification ×200). (A) Control group, (B) model group, (C) sodium hydrosulfide group, (D) DL-propargylglycine.

Evidence of liver injury in the model group, which received an intraperitoneal injection of CCl_4_, was indicated by significantly increased serum expression levels of HA, LN, PcIII, and cIV (Fig. 2), indicative of fibrosis. Pathological damage was evident when compared with the control group. Treatment with PAG resulted in increased serum expression levels of HA, LN, PcIII, and cIV, as compared with the control group, whereas treatment with NaHS resulted in significantly lower serum expression levels of HA, LN, PcIII, and cIV compared with the model group (Fig. 2). The results of HE staining demonstrated that the PAG group exhibited early-onset necrosis and increased inflammatory cell infiltration, as compared with the control group (Figs. 3 and 4). The subgroup treated for four weeks with PAG exhibited severe liver necrosis. The number of blue pixels in the Masson-stained liver sections was measured in order to quantify the amount of collagen fibers. The results of the Masson staining of the control group liver sections suggested a near absence of collagen fibers, whereas treatment with CCl_4_ significantly increased the number of blue pixels in the liver sections, suggesting a large number of collagen fibers were present. Treatment with NaHS resulted in a significantly lower number of blue pixels, whereas treatment with PAG was associated with a significantly higher number of blue pixels, as compared with the control and model groups (Figs. 5 and 6).

### Effects of NaHS and PAG on the mRNA expression levels of CSE and on H_2_S synthesis in rat liver

**Figure 7:**
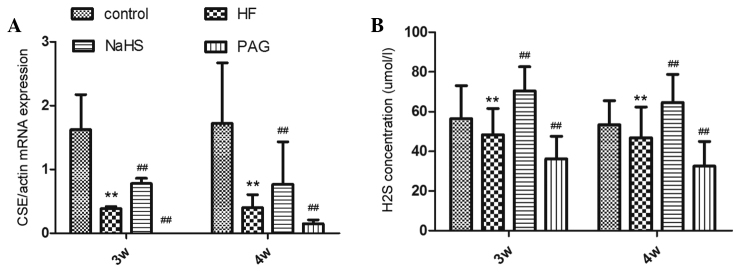
Expression levels of cystathionine-γ-lyase (CSE), hydrogen sulfide (H_2_S) and angiotensin II type 1 receptor (AGTR1) in rats with hepatic fibrosis. (A) The mRNA expression levels of CSE were measured in each group using reverse transcription-quantitative polymerase chain reaction, which demonstrated similar results between week three and four. (B) H_2_S levels were decreased in the DL-propargylglycine group, and conversely increased in the sodium hydrosulfide group, as compared with the control. The error bars indicated the mean ± standard deviation (^**^P<0.01 vs. normal control group, ^##^P<0.01 vs. the model group). HF, hepatic fibrosis.

The gene encoding the H_2_S-forming enzyme CSE was detected in the rat liver, by RT-qPCR of liver RNA. The mRNA expression levels of CSE in the model group were markedly lower, as compared with the normal control group, these results were accentuated by PAG treatment. Conversely, in the NaHS group, CSE content was significantly increased compared with the model group (Fig. 7A). The serum expression levels of H_2_S in the CCl_4_-treated rats were markedly lower than in the normal control group, suggesting that CCl_4_ significantly reduced hepatic H_2_S-producing activity (Fig. 7B).

### Effects of NaHS and PAG on the expression levels of AGTR1 in CCl_4_-induced hepatic fibrosis

**Figure 8:**
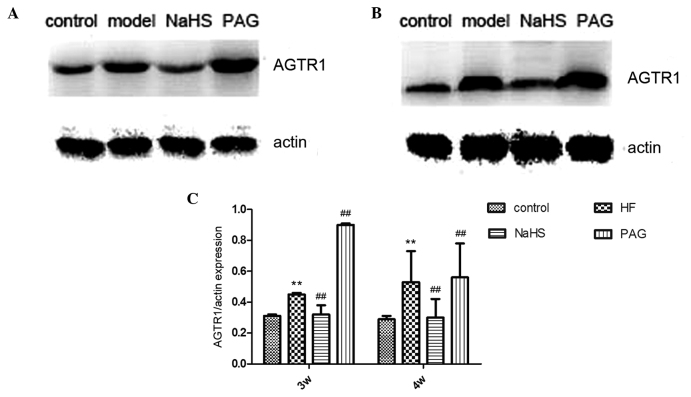
Protein expresssion levels of angiotensin II type 1 receptors (AGTR1) as determined by western blot analysis in the rat groups at (A) week three and (B) week four. Actin was used as an endogenous control. Actin was used as an endogenous control. (C) Quantification of AGTR1 expression levels. The error bars indicated the mean ± standard deviation (^**^P<0.01 vs. normal control group, ^##^P<0.01 vs. the model group). NaHS, sodium hydrosulfide; PAG, DL-propargylglycine.

The hepatic protein expression levels of AGTR1 were evaluated by immunoblot analysis. In the model group, CCl_4_-induced hepatic fibrosis resulted in the significant upregulation of AGTR1 expression, as compared with the normal control group. Conversely, treatment with NaHS resulted in a significant downregulation of AGTR1 expression, as compared with the control group; however, this finding was not significant. PAG treatment significantly increased the expression levels of AGTR1, as compared with the control group (Fig. 8).

## Discussion

CCl_4_ is widely used to induce a chemical model of hepatic fibrosis. CCl_4_ is metabolized to a trichloromethyl radical, leading to increased lipid peroxidation, depletion of glutathione, impaired hepatic anti-oxidant activity, and hepatocyte necrosis (21). Fan *et al* (22) reported that H_2_S administration attenuated hepatic fibrosis and collagen I protein expression in rats exhibiting CCl_4_-induced hepatic fibrosis, inhibited cellular proliferation, and induced cell cycle arrest and apoptosis of activated HSCs. Jha *et al* (23) demonstrated that H_2_S significantly attenuated hepatic I/R injury via preservation of the intracellular redox balance and inhibition of apoptosis during I/R injury. These results suggested that H_2_S may serve as a promising therapeutic agent in the treatment of hepatic I/R injury.

HSCs have a crucial role in the onset of hepatic fibrosis. HSCs express AGTR1 (15), and are activated by the binding of angiotensin II to AGTR1, which in turn leads to the secretion of extracellular matrix components resulting in the development of hepatic fibrosis (24). Activated HSCs also express numerous cytokines, which accelerate hepatic inflammation (24).

Fibrogenesis in chronic liver disease is stimulated by angiotensin II via AGTR1, and may be modulated by angiotensin-converting enzyme inhibitors and AGTR1 antagonists (25,26). In the present study, advanced liver fibrosis was effectively induced by CCl_4_. The results of the present study demonstrated that the protein expression levels of AGTR1 were negatively correlated with the degree of liver fibrosis. Töx *et al* (27) showed that angiotensin II may influence transforming growth factor (TGF)-β-mediated processes via AGTR1, by enhancing Smad2 gene expression in the liver.

Tan *et al* (28) previously investigated the protective role of H_2_S on CCl_4_-induced acute hepatotoxicity, as well as the prophylactic and therapeutic effects of H_2_S on long-term CCl_4_-induced cirrhosis and portal hypertension, mediated by the multiple functions of H_2_S, including antioxidation, anti-inflammation, cytoprotection, and anti-fibrosis. The results of the study indicated that the use of H_2_S may provide potent therapeutic effects against liver cirrhosis and portal hypertension.

The regulation of sinusoidal resistance depends on the aggregation of HSCs around sinusoidal endothelial cells (29). A previous study demonstrated that H_2_S is an autocrine neurotransmitter that is involved in the regulation of HSC contraction and the maintenance of portal venous pressure via K_ATP_ channels (29). H_2_S counteracts impaired vasodilation and HSC contraction, thus reducing portal hypertension in cirrhotic livers (29).

Angiotensin II has been shown to increase the expression levels of hepatic TGF-β1 during the development of hepatic fibrosis (30). Connective tissue growth factor (CTGF) is a hepatic profibrotic mediator, which is a downstream target of TGF-β1 in HSCs (31,32). Tamaki *et al* (33) demonstrated that telmisartan (an AGTR1 receptor blocker) inhibited hepatic fibrosis, induced downregulation of tumour necrosis factor-α, TGF-β1, and CTGF mRNA expression, and reduced the number of α-smooth muscle actin-positive cells in the liver.

In conclusion, the results of the present study demonstrated that H_2_S was able to inhibit liver fibrosis, and hinder the formation of hepatic fibrosis. Downregulation of AGTR1 was closely associated with the progression of liver fibrosis, suggesting that H_2_S may inhibit the expression of AGTR1. The results of the present study contribute to the understanding of the protective effects of H_2_S in liver fibrosis, but whether the mechanisms underlying H_2_S protection, its correlation with angiotensin receptor inhibitors, and its application in human hepatic fibrosis has similar protective effects requires further study.

## Figures and Tables

**Table I:** Number of rats present in each stage of hepatic fibrosis for each group.

Groups	S0	S1	S2	S3	S4
Normal control group	14				
Model group (3 w)			3	4	
NaHS group (3 w)		2	2	3	
PAG group (3 w)			2	5	
Model group (4 w)				3	4
NaHS group (4 w)		1	2	3	1
PAG group (4 w)				2	5

NaHS, sodium hydrosulfide; PAG, DL-propargylglycine; S, stage; w, weeks.
